# Modernising Coeliac Disease Dietitian Follow‐Up: Engagement and Functionality of a Digital Annual Review

**DOI:** 10.1111/jhn.70213

**Published:** 2026-02-08

**Authors:** Octavia Abbott, Cristian Costas‐Batlle, Yvonne Jeanes, Claire Gardiner

**Affiliations:** ^1^ Cambridge University Hospital NHS Foundation Trust Cambridge UK; ^2^ Bradford Teaching Hospitals NHS Foundation Trust Bradford UK; ^3^ British Dietetic Association Birmingham UK; ^4^ School of Health Leeds Beckett University Leeds UK

**Keywords:** annual review, coeliac disease, dietitian, digital, efficiency, virtual

## Abstract

**Introduction:**

UK guidance recommends adults living with coeliac disease (CD) receive annual care from a dietitian. However, many CD provisions have difficulties meeting these demands. The study aimed to evaluate a digital virtual annual review (VAR) tool as an alternative method of reviewing clinically stable adults with CD annually.

**Methods:**

This single‐centre retrospective study analysed data between September 2022 and March 2024. Adults living with CD, diagnosed for > 3 years and deemed clinically stable by a specialist CD dietitian, received a link to complete the digital VAR tool. Chi‐squared analysis assessed the relationship between demographics and engagement with the tool. Median value indicated clinician triage time. Outcomes of the triage process were presented as frequency and percentages. Ethical approval was obtained from local higher education institute, and information governance approval was received through local processes.

**Results:**

The majority (81.7%, *n* = 165) of service users engaged with the digital VAR tool. The demographics did not influence engagement. The specialist CD dietitian required 14 min on average to triage a singular digital VAR tool. Following triage, 72.4% (*n* = 102) did not require interim input prior to their routine annual review. Only 22% (*n* = 31) were deemed to require a follow‐up clinic appointment with the specialist CD dietitian to discuss symptoms or GF dietary adherence. Service users mainly preferred the digital VAR tool for future reviews (55%, *n* = 78).

**Conclusions:**

Our study provides supportive evidence for the effectiveness of a digital annual review tool to improve the efficiency of dietetic service provision for stable adults living with CD.

## Summary

- The majority of service users living with coeliac disease engaged with an annual review that was conducted virtually, using a digital platform irrespective of age, gender and ethnicity.
- The time taken by the specialist coeliac disease dietitian to triage the annual review forms was less than half the time taken for a face‐to‐face clinic appointment.
- Over half of the service users living with coeliac disease preferred a virtual digital approach for future annual assessments.
- Further research is required to understand the barriers and challenges in order to improve the service user experience of using the virtual digital assessment.

## 1 Introduction

Coeliac disease (CD) is a lifelong autoimmune condition activated by gluten consumption in genetically susceptible individuals [1]. Support from healthcare providers is paramount to minimise the risk of poor health outcomes such as low bone density, an increased risk of bowel cancer [2, 3], as well as vitamin and mineral deficiencies [4]. The avoidance of all dietary gluten is the only known treatment for CD. With adequate support from a specialist CD dietitian this restrictive diet can be positively managed to support a healthy lifestyle [1, 5, 6]. Specialist CD dietitians' involvement has been shown to improve adherence to a gluten free diet (GFD) [7, 8, 9, 10], with previous research showing the reduced need for unnecessary repeat endoscopies as an additional result [10]. Service user access to a specialist CD dietitian is recommended in international [11, 12] and national [13, 14] guidance. Review clinics involving both dietitians' and gastroenterologists' expertise are considered the best standard of care [13]. However, a recent UK survey reported dietetic gastroenterology services were inconsistently available across NHS trusts, let alone a specialist dietetic CD service, with less than 50% of dietetic gastroenterology departments offering a dedicated CD service provision [15]. This lack of dedicated CD provisions makes providing optimal care to adults living with CD challenging, in addition to not meeting national guidance recommendations for providing annual reviews [13]. This was confirmed by a recent UK‐based survey where 60% of service users reported not receiving annual reviews [16]. As a result, service user experience has been impacted considerably, affecting their reported quality of life [17], which raises concern for a long‐term health outcome particularly in vulnerable service users who want the support [18]. Notably individuals with CD may require differing levels of support to achieve and maintain long‐term management of their condition. Adopting varied models of care beyond the individual in‐person consultation, such as Artificial Intelligence‐enabled pathways and alternative digital approaches may enable Trusts to tailor the intensity of support to individual needs promoting both effective disease management and greater service‐user responsibility for their own healthcare management [19].

The future NHS 10‐year plan [19] prompts the consideration of alternative methods of conducting consultations. Currently in CD care, in‐person, telephone, group education sessions or online approaches are offered by a health care professional depending on the UK location. However, the most effective approach for reviewing CD service users is still being explored. Stuckey [20] reported that engagement with a virtual approach was substantial, with 91.7% (*N* = 246) of the service users living with CD completing self‐directed questionnaires. In contrast, other studies have reported reduced engagement with digital assessments, citing barriers such as limited opportunities to ask questions [21, 22]. Nonetheless, in‐person consultations also present challenges, including the time taken to attend, travel time and parking costs potentially hindering attendance [23].

With an expanding CD population and limited in‐person and telephone clinic capacity, a CD dietetic service evaluated their service provision for annual review appointments to explore opportunities of remotely reviewing service users using a digital tool. The aim of this study was to explore the relationships between demographics and engagement with a digital virtual annual review (VAR) tool to identify potential service access inequalities. A secondary aim was to evaluate the efficiency of the digital VAR tool on the dietitian's review duration and decision time.

## 2 Methods

### 2.1 Study Design

This retrospective study analysed routinely collected data from a dietitian‐led CD service in an NHS trust in the United Kingdom. Service provision was comprised of two specialist dietitians with expertise in CD and a gastroenterologist. The study received University ethical approval for an undergraduate final year research project, and information governance approval was received through local NHS trust procedures. The study has been developed in line with strengthening the reporting of observational studies in epidemiology (Appendix S1)

The digital VAR tool (Appendix S2) is an assessment questionnaire developed by a specialist CD dietitian leading a CD provision, informed by clinical expertise and published literature. The tool was reviewed by dietitians, gastroenterologists, and adults living with CD before use. The tool assesses CD symptoms, adherence to the GFD, quality of life, service user satisfaction, and consent for data use in further research.

To ensure the largest sample size, all known adult service users under the care of the dietitian‐led CD clinic and living with CD for > 3 years, as well as being deemed stable in their condition received the digital VAR tool as part of their routine care. Service users were deemed stable due to the absence of CD‐related symptoms, adequate GFD adherence levels and adequate response to the GFD on last review. In instances where the services user was unable to complete the digital VAR tool, family members and carers had the option to complete it on their behalf. Where this was not possible, alternative options for the annual review were provided, such as one‐to‐one in‐person or telephone clinics. The digital VAR tool was implemented to replace an in‐person or telephone annual review appointment with service users who had consented to this in their previous annual review. The digital VAR tool was distributed via the DrDoctor platform, which is a secure digital engagement system used in healthcare settings [24] between 30th September 2022 and 30th March 2024. A confidential secure link to complete the digital VAR assessment was delivered to the service user via text message. Upon completion, responses were immediately accessible to the dietitians to triage. The dietitians then reviewed and triaged the responses flagging any that raised clinical concern, such as recent unexplained weight loss of > 10%, issues with GFD adherence via which foods service users were eating, and new digestive or non‐digestive symptoms impacting the service user. Dietitians documented clinical findings and follow‐up actions in the service user's electronic patient record (EPR). Service users who did not respond to their link to complete their digital VAR tool received a letter offering the option of an administrator aiding completion over the phone or, depending on preference, a clinic appointment.

### 2.2 Data Collection

**Figure 1:**
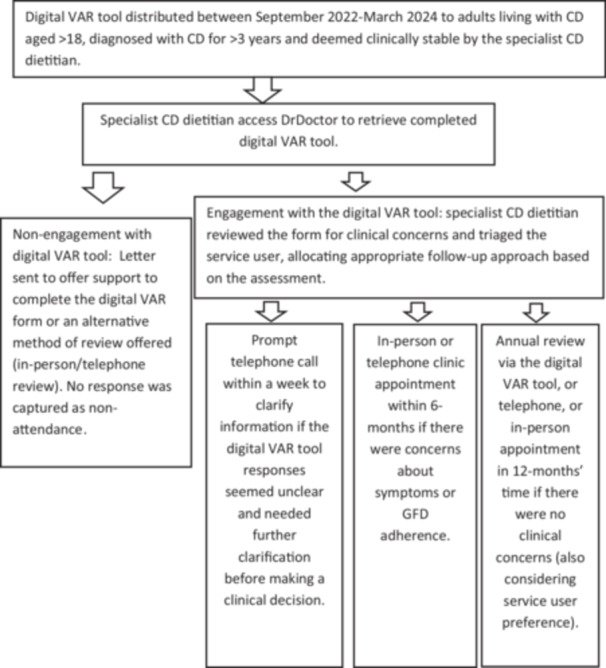
Service process workflow: Distribution of questionnaire to actions taken.

EPR, a digital software program used to store the service users' medical information, was accessed to gather routinely collected data including biological sex, age, and length of time diagnosed with CD for all service users. The digital VAR tool was accessed to collect data regarding individuals' ethnicities. Data was not accessed for those who opted out of the research via the VAR tool. To ensure anonymity service users ages were grouped in large bands and dichotomous groups were created with ethnicity (White and Ethnic Minorities) due to limited numbers for some of the original ethnicity groups used. Actions recorded from the triaging process of the completed VAR tools were also obtained for EPR. The completed digital VAR tools provided information about symptoms, GFD adherence, service users' satisfaction and preferred methods of future follow‐up. The time taken to review and triage a digital VAR tool, which included any telephone calls made to clarify answers and any emails sent with further information offering support, as well as documentation, was also recorded on an excel spreadsheet between 30th October 2023 and 30th March 2024. Figure 1 shows a step‐by‐step approach to the data collection process.

### 2.3 Statistical Analysis

Data analysis utilised SPSS statistical package version 29 (IBM.Corp). The demographics and engagement data were grouped and presented as percentages and numbers. Chi‐squared test was used to explore if there was a relationship between biological sex, age, ethnicity or length of time diagnosed with CD and engagement with the digital VAR tool. The *p*‐value < 0.05 noted significant difference [25]. Data on triage outcomes and time taken to triage was presented as frequencies, with the average time expressed as a median and interquartile range (IQR).

## 3 Results

### 3.1 Engagement

Out of 202 digital VAR tools, 81.7% of service users engaged (*n* = 165); however, 24 service users opted out of their data being used in research. Of the 141 service users who engaged and consented to data usage, 12 required aids from a family member to complete the digital VAR tool and 1 digital VAR tool was completed by the aid of an administrator. The majority of service users who engaged with the digital VAR tool were female (*n* = 101) and aged 41–51 years (*n* = 41). Over 50% of the service users identified as white (*n* = 82), and most service users had been diagnosed with CD for 3–6 years (*n* = 45). However, no demographic characteristics were found to significantly impact the engagement with the digital VAR tool (Table 1).

**Table 1:** Service user engagement with the VAR tool, based on reported demographics and length of time diagnosed with coeliac disease.

Category	Subcategory	VAR tool engagement *N* (%)	VAR tool non‐engagement *N* (%)	*p* value^a^
Total		141	37	
Age group (years)	18–40	36 (26)	16 (43)	0.11
	41–62	71 (50)	14 (38)	
	> 63	34 (24)	7 (19)	
Biological sex	Male	40 (28)	13 (35)	0.42
	Female	101 (72)	24 (65)	
Ethnicity	Ethnic Minorities	53 (38)	19 (51)	0.27
	White	82 (58)	16 (43)	
	Prefer not to say	6 (4)	2 (5)	
Length of time diagnosed with CD (years)	3–10	84 (60)	28 (76)	0.20
	11–14	20 (14)	3 (8)	
	15+	37 (26)	6 (16)	

^a^ Chi‐squared.

### 3.2 Outcomes of the Digital VAR Tool

Following review and triage of the digital VAR tool, 72.4% (*n* = 102) of service users who engaged did not require any immediate follow‐up, therefore requiring a routine annual review in 12 months. A small number of service users 5.7% (*n* = 8) required a prompt telephone call within a week to clarify information provided on the VAR tool before making a clinical decision. Finally, 22% (*n* = 31) were deemed to need a follow‐up clinic appointment with the specialist CD dietitian within 6 months to further discuss symptoms or GFD adherence.

**Figure 2:**
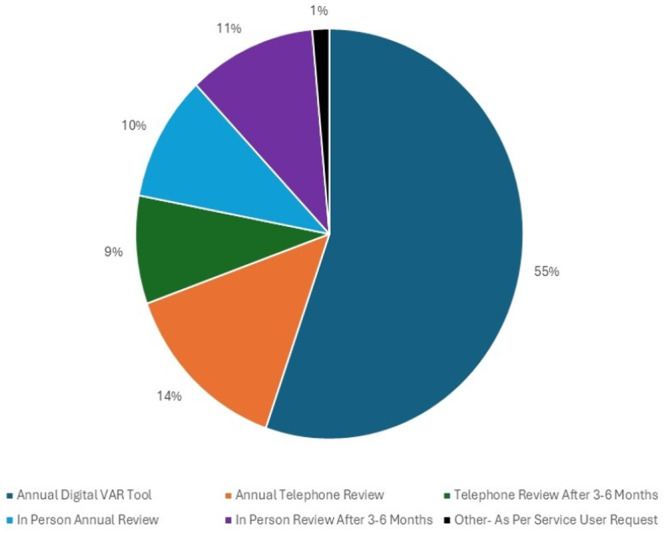
Service users review preference for their next appointment.

There was an opportunity for all service users to state their preferred mode of future dietetic contact for their next review (Figure 2). The majority (55%, *n* = 78) were happy to be reviewed using the digital VAR tool for their next annual review. In total, 21% (*n* = 29) wanted to be reviewed in‐person; however, 11% (*n* = 15) wanted a review within 3–6 months rather than an annual review. A telephone review was deemed acceptable by 23% (*n* = 32); however, 9% (*n* = 12) requested this to be within 3–6 months.

### 3.3 Case Review and Decision‐Making

The specialist CD dietitian noted how long it took to triage and act on the information from the 63 of the 141 the VAR tool responses submitted between 30th October 2023–30th March 2024. Out of the 63 VAR tools this was measured for, the median triage time was 14 min (IQR: 10.5–15 min).

## 4 Discussion

Our study reports the engagement and efficiency of a novel digital tool when caring for clinically stable adults living with CD at the annual review stage. It is also the first study to evaluate such a tools functionality in terms of time efficiency to help inform clinicians of the potential value it has. There is limited research investigating alternative digital ways of reviewing adults living with CD at the annual review stage, that also explore demographic engagement and time efficiency for clinicians.

Our study showed that when providing an option to complete an online annual review, most service users engaged with this approach (81.7% *n* = 165), irrespective of their demographics. This is of interest as existing research has found older adults engage less with digital technology [26]. However, the acceptability of a digital approach to healthcare in relation to age does appear to be changing. A more recent study by Williams et al. [27] found 37% of viewers of an online webinar providing dietary advice for IBS were over the age of 55 years. This can be comparable to our study, which observed 24.1% (*n* = 34) of digital VAR engagers being > 63 years old. However, Williams et al. [27] study was carried out during 2020 when the United Kingdom was intermittently locked down, so results may have been influenced by a national push for digitalisation. Alternatively, the findings from both studies may also show a change in attitude regarding age and accessing online healthcare since the pandemic. Our study discovered 18.3% (*n *= 37) of service users did not engage with their digital VAR tool, and a small percentage of service users (7.5%, *n* = 13) required support to aid completion. Exploring the reason for non‐engagement or need for support was not within the scope of this research. However, a lack of motivation or confidence in following a GFD [28], as well as challenges encountered, such as associated with language barriers and limited access to technology could be considered [29].

Our findings suggest the digital VAR tool was time effective for the dietitian, saving on average 16 min including administration time, per service user (*n* = 63), when comparing this to the traditional 30‐min one‐to‐one consultation. There is likely to be a trade‐off with this process taking less time, the more familiar the dietitian is with the process. The finding that 72.3% of service users did not require interim follow‐up, before their next annual review in 12 months, suggests it is an effective and efficient method of reviewing clinically stable adults living with CD, benefiting the individual as well as saving dietitian time. This finding also supports the concept that not every service user with CD requires the same level of dietetic input on a yearly basis. In addition, this study suggests cost savings in terms of dietetic time, which can also translate to generating more clinic slots for new and symptomatic service users, as well as reducing the need for physical resources such as clinic space. The service user will also benefit not having to travel to the hospital, which aligns well with the aims of the NHS 10‐year health plan [19]. However, the modest sample size when addressing the duration of time it took to triage a digital VAR tool (*n* = 63) may influence the validity of these results.

We did not explore how long it took the service users to complete the digital VAR tool. This data would have been insightful for future digital VAR tool users; however, we did not see any feedback that suggested the form took an adverse amount of time to complete, as within the form service users had an opportunity to highlight issues with the form. The findings showed 55% of service users were happy to undergo future reviews via the digital VAR tool indicating that service users likely did not find filling in the form too burdensome.

The finding of 72.3% of service users not requiring interim follow‐up prior to their existing annual review suggests the tool may be an acceptable and preferred way to review clinically stable adults living with CD. However, we did not compare whether this proportion differed to our in‐person or telephone annual review consultations. This may be helpful to explore in future.

It was of interest that a high percentage of the cohort engaged and wanted future reviews conducted in the same way, as this differs to findings of similar studies [21, 30]. High engagement in our study could be reflective of familiarisation; this cohort had been using digital forms for the past 2 years on the same software platform as part of their routine care. However, this high engagement could also show an increasing shift over time in service users' perspectives to virtual healthcare since the Covid‐19 pandemic, which recent evidence has found [31, 32]. However, this is not a consistent finding as Trott et al. [21] found digital applications were least preferable, with only 38% of service users choosing this. This contradiction in findings could suggest the high engagement was due to familiarity or that there are other contributing variables at play within or cohort that we are not yet aware of.

Existing research [33, 34] observed that factors such as socio‐economic status, access to technology and individuals' digital literacy can have an impact on engagement to digital healthcare with one study finding service users from a lower household income preferred in‐person appointment [35]. Although we did not collect data on socioeconomic background in this study, the service users do reside in what is recognised as a deprived region of the United Kingdom. The study in question compared in‐person, telephone and online videos but did not include digital questionnaires in the study design, so the studies may not be directly comparable. These contradictory findings could also suggest that appointment style preference may vary between locations, and there are likely a number of contributing factors to consider rather than household income alone. The high engagement rate implies our service users had substantial access to technology and that the majority possessed adequate levels of digital literacy.

To gain a better understanding and to ensure digital literacy did not have an impact on engagement, further research should acknowledge why 45% of service users did not want to undertake future reviews via the digital VAR tool. Of particular interest was ethnicity as existing literature is conflicting to the findings in our study as often a poorer engagement [36, 37]. Albeit, these findings often reflect the impact of underlying variables such as the financial or socioeconomic status of the service user. Nonetheless, further exploration is suggested to identify whether the digital questionnaire could be made more culturally appropriate and accessible for retrospective demographic groups. Further research into why service users did not choose the digital VAR tool for future assessments should be conducted, as research has identified the psychological impact of living with CD, which is becoming increasingly more evident [38]. It is possible that a service user may have opted for an in‐person appointment for their next annual review for support and reassurance.

One of the key strengths and unique aspects of this research is the digital VAR tools self‐reporting nature. Self‐reporting digital questionnaires are cost‐effective for the Trust [39], due to not requiring a present clinician or a clinic room, as well as being beneficial for the service user due to not having to travel to appointments and being able to complete it at their convenience. With the nature of questionnaires there is the risk service users may provide incomplete information [40], although other healthcare studies have found individuals can be more willing to disclose sensitive behaviours and health information on digital approaches to care, such as questionnaires [41]. Rickwood and Coleman‐Rose (2023) [42] suggest that self‐reported data elicits greater disclosure of sensitive information due to a reduced pressure to conform to perceived healthcare provider expectations. Therefore, there is a possibility that service users are more honest about their gluten consumption on the digital VAR tool than in‐person or via telephone consultation. In addition. A digital tool lends itself well to incorporating validated adherence questionnaires like the CDAT [43] or Biaggi adherence test [44], which could help to assess adherence in a more standardised and validated way, though it is acknowledged that these questionnaires still have limitations. An interesting and direct comparison could also be drawn by looking at GFD adherence responses on the digital VAR tool compared to in‐person clinic appointments to provide further results. In our study, where clarity on the information provided was needed, the dietitian contacted the service user for further information before making a clinical decision.

An experienced CD dietitian alongside service users and gastroenterologists created a questionnaire for this study ensuring it was reflective of routine practice of in‐person and telephone appointments, as well as being culturally relevant. However, as this is a new approach in CD care, this method would benefit from validation in the future to ensure the information collected is accurate, appropriate and consistent. It could also integrate questions or full questionnaires from existing GFD adherence forms, such as CDAT [43] and Biaggi [44] adherence tests to help accuracy and standardisation. This could help other CD services to use a standardised approach in the annual review of clinically stable adults living with CD, which would likely be beneficial given this study's high engagement rate. A structured approach with a validated questionnaire could further save dietitian time as it may allow scope for other HCP's such as dietetic assistants to analyse the information, and provide pre‐defined responses, with the additional option of emerging AI being able to help automate decision making in the triage process too in simple cases. In addition, it is also worth considering that this type of review method could have application in other stages of CD follow‐up or in other medical conditions if it were adapted accordingly. A similar questionnaire can help triage service users upon referral to CD services, or screen service users at different points to identify who may need earlier appointments, supporting a more efficiently run service, whilst acknowledging not all service users will require the same level of support and keeping service users engaged with their care.

Although our study has found positive results in terms of service user engagement and time efficiency due to its retrospective design, it has not looked at service users' satisfaction in depth, and it would be insightful for future research to explore this. In other health conditions, such as diabetes virtual approaches to healthcare have been integrated and have identified several factors which increase service users' satisfaction, such as: time saving and efficiency [45]. Conducting further research into time efficiency of the digital VAR tool for service users' satisfaction, would also aid in establishing if service user satisfaction is being met in different ways.

## 5 Conclusion

In a growing cohort of service users where the requirement of medical and dietetic annual reviews is recommended annually, our study provides evidence supporting the digital VAR tool as an effective alternative for reviewing clinically stable adults with CD. The high engagement amongst service users along with the preference to use this approach in the future suggests it may be a method to consider for reviewing adults living with CD deemed clinically stable. It would be valuable to validate the digital VAR tool to allow its utilisation by other hospitals and to explore whether similar findings emerge in additional services when using this digital tool. This study may serve as a foundation for further research into the effectiveness of using increasingly sophisticated technologies to both enhance service users experience and the efficiency of CD services, whilst also providing insights to inspire other services that support users with alternative long‐term health conditions.

## Author Contributions

Octavia Abbott, Cristian Costas‐Batlle and Claire Gardiner designed the study. Octavia Abbott collected, processed, and ran the statistical analysis on the data. Critical feedback and guidance to the final manuscript version was provided by Claire Gardiner, Cristian Costas‐Batlle and Yvonne Jeanes. All authors agreed on the definitive version of the paper submitted to publication.

## Funding

The authors received no specific funding for this work.

## Ethics Statement

Ethical approval via the UK Health Research Authority was not required for this study because it was deemed an evaluation of the dietetic service. Local approval was given by both Research and Development and Information Governance departments at Bradford Teaching Hospitals NHS Foundation Trust [Reference number: 2023IG0039], as well as receiving ethical approval from Leeds Beckett University.

## Conflicts of Interest

C.C.B. is a consultant to Takeda Pharmaceuticals.

## Transparency Statement

I hereby declare that this research is my own original and uncopied work, and I have given full acknowledgement to all cited and referenced sources used. This research is based off, but rewritten, a piece of work submitted as an undergraduate degree at Leeds Beckett University.

## Supporting information

Appendix 1.

Appendix 2.

## Data Availability

The data is not publicly available due to privacy and ethical restrictions. However, the data to support this study findings can be requested from the corresponding author.
