# Supplementary material for: Modernising Coeliac Disease Dietitian Follow‐Up: Engagement and Functionality of a Digital Annual Review
Source: J Hum Nutr Diet. 2026 Feb 8;39(1):e70213. doi: 10.1111/jhn.70213 (PMC12884136; doi:10.1111/jhn.70213)
Supplement: Supplementary file 2 — Appendix 2. [file JHN-39-0-s002.docx]

Appendix 2

Patient Form response

Coeliac Disease Virtual Annual Review

Completing this assessment will help our specialist coeliac disease dietitian assess you remotely. This form temporarily replaces an annual clinic appointment.

The digital form can help monitor all patients in a timely manner whilst also highlighting any issues, which will enable the right support to be accessed when needed.

The form will take approximately 10 minutes to complete.

---

Who is completing this form? *
I am the patient
I am completing this form on behalf of the patient

---

Weight

1. Have you had any unexplained weight loss in the last 6 months? *
Yes
No

2. Have you weighed yourself recently? *
Yes
No

2.1 Approximately when did you weigh yourself? *
Thursday, 27th June

2.2. Please specify a unit of measure to specify your weight: *
Kilograms (kg)
Stones and pounds (st, lbs)

2.2.1 Kilograms (kg): *
70.0

3. Do you have any concerns with your weight? *
Yes
No

---

Vitamins and Minerals

1. Are you taking any vitamins and/or minerals? (e.g. iron, vitamin D, folic acid, calcium) *
Yes
No

1.1 Are any of these vitamins and/or minerals prescribed by your GP? *
Yes
No

1.2 Please specify which vitamins and what dose *
One tablet 1000mg/800IU

---

Current Symptoms Assessment
Are you experiencing any symptoms that you think could be linked to your coeliac disease or that you want to let us know about? *
Yes
No

---

Bowel Assessment

1. With regards to your bowels... *
I am opening them more often than I would like
I am not opening them as often as I would like
I have no issues with how often I open them

2. How often do you currently pass bowel action? (you can select more than one) *
Once every 7 days
Once every 4-6 days
Once every 2-3 days
Once a day
2-3 times per day
4-6 times per day
7 or more times per day
I have a stoma bag

3. Which of the above images represents your typical stool type? *
Type 1 - Separate hard lumps, like nuts (hard to pass)
Type 2 - Sausage-shaped but lumpy
Type 3 - Like a sausage but with cracks on its surface
Type 4 - Like a sausage or snake, smooth and soft
Type 5 - Soft blobs with clear-cut edges (passed easily)
Type 6 - Fluffy pieces with ragged edges, a mushy stool
Type 7 - Watery, no solid pieces. Entirely Liquid

4. Have you recently had any blood in your stools? *
No
Yes

---

Gluten Free Diet

1. To your knowledge, are you following a strict gluten free diet? *
Yes
No

2. Over the past 4 weeks, how many times have you eaten food containing gluten knowingly? *
0
1-2
3-5
6-10
+10 times

3. Do you read food labels when shopping for gluten free food? *
Yes
No
No because I only buy foods that are labelled as gluten free

3.1 To your knowledge, which of these ingredients can contain gluten when reading a food label? *
Soya
Maize
Wheat
Barley
Rye
Oats
Milk
Yeast

3.2 With regards to oats... *
I eat regular oats that are not gluten free
I only eat gluten free oats
I don’t eat any oats

4. How confident are you with reading a food label to know if the food contains gluten? (1 = Not confident at all, 5 = Extremely confident) *
1
2
3
4
5

5. How often do you eat foods from food packets that don’t have gluten in the ingredients but say “made in a factory with gluten” or “may contain gluten”? *
Never
Rarely
Sometimes
Very often
Always

6. Do you eat crisps? *
Yes
No

7. Do you eat chocolate? *
Yes
No

7.1 Which brands of chocolates do you eat? *
Bounty
Kit-Kat
Maltesers
Mars
Snickers
Twirl
Wispa
Other: Asda dark

8. Are you member of Coeliac UK? *
Yes
No

8.1 Do you use their resources to ensure the food labels you check are gluten free? *
Yes, I use the Gluten Free Food checker app
Yes, I use the Food and Drink Directory Book
No I don't use their resources to check food labels. I just read the label

9. How often do you make sure gluten free food is prepared separately? *
Never
Rarely
Sometimes
Very often
Always

10. When you eat out or order food in, do you tell the person who is cooking about your coeliac disease? *
Never
Rarely
Sometimes
Very often
Always

10.1 Do you also ask about gluten cross-contamination in the kitchen? *
Yes
No

---

Gluten Free Diet - Quality of Life

How difficult do you think it is to follow the gluten free diet at present? (1 = Not difficult at all, 5 = Extremely difficult) *
1
2
3
4
5

How confident are you in managing your coeliac disease through diet? (1 = Not confident at all, 5 = Extremely confident) *
1
2
3
4
5

---

Follow-Up

1. Do you have any other concerns you would like to discuss with your specialist dietitian? *
Yes
No

2. How would you like to be followed-up going forward? *
I am happy to have another virtual annual review
I would like a telephone appointment for my annual review next year
I would like a face-to-face appointment for my annual review next year
I would like a telephone appointment in the next 3-6 months
I would like a face-to-face appointment in the next 3-6 months
Other

3. Are you happy to be followed-up by the coeliac dietetic service using this virtual form next year as well, and then a telephone review in the third year? *
Yes
No

4. Do you have any concerns or anything you would like to let the team know? *
No

---

Feedback about the Coeliac Service

How satisfied are you with the coeliac service so far?
1
2
3
4
5

Please explain why you chose the above rating
Virtual review is good

What do you think could be improved about this coeliac service?
The updates on new products are very similar to the coeliac UK so maybe a local slant could be added?

---

Ethnicity

What is your ethnicity if you are happy to share it? *
I prefer not to share it
White
Mixed or multiple ethnic groups
Asian or Asian British
Black, African, Caribbean or Black British
Arab
Other

Please specify: *
English, Welsh, Scottish, Northern Irish or British
Irish
Gypsy or Irish Traveller
Any other White background

---

Feedback questions

Would you be happy for us to use this data anonymously for research? *
Yes
No

How easy did you find it to use this form? (1 = Very easy, 5 = Very difficult)
1
2
3
4
5

Do you have any additional comments/feedback about this form?
Not as of now

Would you like us to share some resources that can help with the gluten free diet once you submit the questionnaire?
Yes
No
